# The College

**Published:** 1854-07

**Authors:** 


### EDITORIAL DEPARTMENT.

---

### THE COLLEGE.

The Faculty have determined to change the time of the opening of the College Course for next winter to the first of November and close about the first of March. For various reasons we have concluded to make the change and we believe it will prove more satisfactory to all concerned. Circulars will be issued soon giving particulars, to which, for further satisfaction, our readers are referred.

We would remark by the way for the gratification of the friends of the School, that the secret and undercurrent efforts to distract and break down the College have most *signally failed*, as all such disgraceful means will in the end do. We have not room now to dwell upon the particulars of this *dark plot*, but will refer to it again in the future. We take this occasion to thank our numerous friends over the State who were kind enough to put us in possession of the designs and the means employed, and also for their expressions of friendship and promises of aid if required.

From present appearances there will be a larger class than last session, perhaps double, and we would here assure those who have determined to attend the coming session, that the Faculty are determined to make it still more interesting and profitable, and that it were well for those to make their arrangements to that effect at as early a day as possible.

